# Current and New Approaches in GMO Detection: Challenges and Solutions

**DOI:** 10.1155/2015/392872

**Published:** 2015-10-15

**Authors:** Marie-Alice Fraiture, Philippe Herman, Isabel Taverniers, Marc De Loose, Dieter Deforce, Nancy H. Roosens

**Affiliations:** ^1^Platform of Biotechnology and Molecular Biology (PBB) and Biosafety and Biotechnology Unit (SBB), Scientific Institute of Public Health (WIV-ISP), J. Wytsmanstraat 14, 1050 Brussels, Belgium; ^2^Technology and Food Sciences Unit, Institute for Agricultural and Fisheries Research (ILVO), Burg. Van Gansberghelaan 115, Bus 1, 9820 Merelbeke, Belgium; ^3^Laboratory of Pharmaceutical Biotechnology, Faculty of Pharmaceutical Sciences, Ghent University, Ottergemsesteenweg 460, 9000 Ghent, Belgium; ^4^Department of Plant Biotechnology and Bioinformatics, Faculty of Sciences, Ghent University, Technologiepark 927, 9052 Ghent, Belgium

## Abstract

In many countries, genetically modified organisms (GMO) legislations have been established in order to guarantee the traceability of food/feed products on the market and to protect the consumer freedom of choice. Therefore, several GMO detection strategies, mainly based on DNA, have been developed to implement these legislations. Due to its numerous advantages, the quantitative PCR (qPCR) is the method of choice for the enforcement laboratories in GMO routine analysis. However, given the increasing number and diversity of GMO developed and put on the market around the world, some technical hurdles could be encountered with the qPCR technology, mainly owing to its inherent properties. To address these challenges, alternative GMO detection methods have been developed, allowing faster detections of single GM target (e.g., loop-mediated isothermal amplification), simultaneous detections of multiple GM targets (e.g., PCR capillary gel electrophoresis, microarray, and Luminex), more accurate quantification of GM targets (e.g., digital PCR), or characterization of partially known (e.g., DNA walking and Next Generation Sequencing (NGS)) or unknown (e.g., NGS) GMO. The benefits and drawbacks of these methods are discussed in this review.

## 1. Introduction

With the aim to improve the agricultural practices and nutritional quality, plant breeding techniques have been developed to produce genetically modified (GM) crops expressing interesting traits such as herbicide tolerance, insect resistance, and abiotic stress resistance [1]. To this end, new combinations of their genetic material are created through the use of modern biotechnology [2]. The first genetically modified organism (GMO) approved for the commercialization was the Flavr-Savr tomato in 1994. From that time, 181.5 million hectares of planted GM plants in 28 countries were reported in 2014 [1]. Given that the “right to know” for the consumers, GMO labeling policies have been established in several countries around the world with a threshold of tolerance varying between 0 and 5%. Therefore, the presence of GMO in the food/feed chain is controlled by the competent authorities [3]. To guarantee the GMO traceability, a key factor in the implementation of these regulations, several strategies, categorized as indirect (protein-based methods) or direct (DNA-based methods), have been developed to detect GMO in food/feed samples by using different technologies. Among the protein-based approaches, which target proteins encoded by the transgenes, several methods depend on the Enzyme-Linked Immunosorbent Assay (ELISA) technique (Table 1) [4–21]. A portable immunoassay system was also proposed (Table 1) [22]. As an alternative, the immuno-PCR method was used to identify GMO (Table 1) [23, 24].

**Figure 1:**
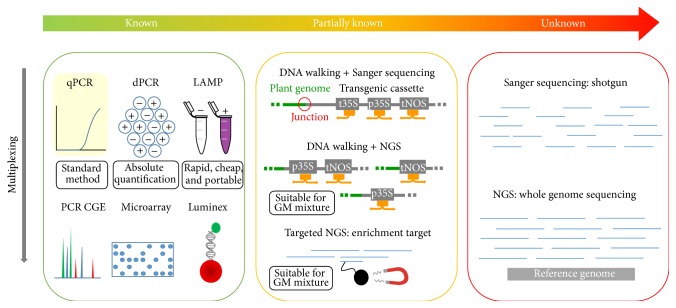
Suitable application of GMO detection approaches regarding the adopted strategy as well as the available information about the sequences of tested GMO.

Furthermore, protein-based methods include the use of the mass spectrometry-based technology as a tool allowing characterizing GM crops [25]. However, although they present several advantages such as the rapidity and simplicity, the protein-based methods depend on the expression level of targeted proteins, which is variable according to the plant tissues and the plant developmental status. Moreover, the proteins are highly degraded or denatured by food processing. Any modification in the targeted proteins could indeed alter the specificity and sensitivity of the assay. In addition, this strategy is not applicable if the genetic modification has no impact at the protein level [26, 27]. To overcome these issues, many DNA-based methods, targeting straightforward transgenic integrated sequences, have been widely developed. Even if quantitative PCR (qPCR) is the method of choice in GMO routine analysis, its inherent PCR properties imply some limitations. Therefore, to address these challenges, some alternative approaches have been developed, allowing notably providing faster detection of GM targets individually amplified in both routine laboratory and field (e.g., loop-mediated isothermal amplification (LAMP)), simultaneous detection of several GM targets (e.g., PCR capillary gel electrophoresis (CGE), microarray, and Luminex), more accurate quantification of GM targets (e.g., digital PCR (dPCR)), or characterization of partially known (e.g., DNA walking and Next Generation Sequencing (NGS)) or unknown (e.g., NGS) GMO (Figure 1). These DNA-based approaches and their targets are described in this review. In addition, the most appropriate uses of these approaches are discussed according to the adopted strategy of GMO detection as well as the available information about the sequences of tested GMO.

## 2. GMO Detection Approaches

### 2.1. qPCR Technology

The qPCR system, which is the most common strategy, allows detecting, identifying, and quantifying GMO via the SYBR Green or TaqMan chemistries (Figure 1) [28]. Using a primer pair specific to the target, these qPCR chemistries are both based on PCR amplification recorded in real time with the fluorescence originated either from the asymmetrical cyanine dye binding to double-stranded DNA (SYBR Green) or from the fluorogenic probe specific to the targeted sequence (TaqMan) [29]. This technology is suitable for both unprocessed and processed food/feed matrices since amplicons of around 100 bp are usually amplified. Even if numerous qPCR methods have been reported, three main steps are typically followed in GMO routine analysis [30]. First, the potential presence of GMO is assessed via a screening approach targeting the most common transgenic elements found in GMO, such as p35S (35S promoter from cauliflower mosaic virus) and tNOS (nopaline synthase terminator from* Agrobacterium tumefaciens*). In addition, some markers more discriminative, such as Cry3Bb, gat-tpinII, and t35S pCAMBIA, and taxon-specific markers could also be used. This step allows establishing a list of the potential GMO present in the tested samples and preventing further unnecessary assays in the subsequent steps (Table 2) [28, 30–34]. Several of these screening markers are validated, based on minimum performance requirements, at the EU level following ring trials and are included in the Compendium of reference methods for GMO analysis [35]. According to the positive and negative signals observed for the different screening markers tested, GM events potentially detected are in a second step identified using construct-specific or event-specific markers targeting, respectively, the junction between two elements inside the transgenic cassette or the junction between the transgenic cassette and the plant genome. In order to properly discriminate each GM event, the event-specific markers are currently favoured since the unique transgenic integration sites are targeted. Finally, the amount of identified GM events present in the tested food/feed samples is determined. Using event-specific and taxon-specific markers, this quantification step is carried out on the basis of the number of copies belonging to the transgene and to the endogen (Table 2) [30]. All the methods used to identify the EU-authorized GMO as well as the GMO for which the authorization is pending or is subjected to be withdrawn in the case of low level presence (LLP) have been provided by the applicants and are reported in the Compendium of reference methods for GMO analysis [35]. In combining several taxon-specific, event-specific, and construct-specific TaqMan markers in a 96-well prespotted plate, a real-time PCR based ready-to-use multitarget analytical system has been developed to allow the simultaneous identification of thirty-nine GM events [36].

In spite of its flexibility, simplicity, rapidity, and high analytical sensitivity, especially crucial to detect a low amount of GM targets, the success of the qPCR strategy depends however on some factors. For instance, the throughput of the qPCR strategy is usually limited to one marker per reaction. Due to the increasing number of GMO, additional markers have continually to be developed and used to fully cover their detection, which could thus make the laboratory work and the analysis of the results quite complex and laborious [32]. In addition, this a priori approach targets only known sequences. Therefore, negative signals guarantee only the absence of known GMO in the tested food/feed samples. Similarly, in case of unexplained signals, in other words, the obtaining of positive and negative signals that found no correspondence with known GM events, the presence of unknown GMO could only be suspected. Indeed, the detection of GMO by qPCR is notably based on transgenic elements originated from natural organisms, such as p35S from CaMV and tNOS from* Agrobacterium*. For this reason, the qPCR system provides merely an indirect proof of the presence of GMO in a food/feed matrix since it could only be confirmed by the sequence of their transgene flanking regions. Concerning the quantification step, its achievement depends on the availability of Certified Reference Materials (CRM) [30, 33, 125]. Finally, the presence of inhibitors, such as polysaccharides, polyphenols, pectin, xylan, or fat, could alter the efficiency of the PCR reaction. Consequently, a later qPCR signal than theoretically expected will be observed, inducing an underestimation or even concealing the amount of GMO present in the tested sample [126–128].

#### 2.1.1. qPCR Analysis Tools

In order to facilitate the interpretation of results, rapid and cost-efficient systems have been developed via analytical tools integrating simultaneously several targets. To this end, the CoSYPS platform (Combinatory SYBR Green qPCR Screening), which is a decision support system (DSS) at the screening level, has been successfully developed. For each tested food/feed matrix, this DSS combines immediately the experimental *C*_*t*_ and *T*_*m*_ values obtained with the twenty SYBR Green methods, running in a single 96-well plate and targeting plant gene, taxon genes, and transgenic elements (Table 2). This selection of screening markers allows both covering at least all the EU-authorized GMO and LLP cases (e.g., with p35S and tNOS) and, as far as possible, discriminating between themselves and some EU-unauthorized GMO (e.g., with t35S pCAMBIA and gat-tpinII) in order to reduce the number of identifications/quantifications to carry out downstream [30, 33, 34, 129]. An alternative to interpret qPCR results is provided by the GMOseek and GMOfinder databases, containing reliable information on GMO. Following the interpretation of the experimental results, obtained with in-house or EU reference methods, the names of positive elements are introduced in the databases to provide a list of potentially detected GMO that will be then experimentally verified [130, 131]. The truthfulness of these predictions is however diminished since elements identically named can possess different sequences and the detection methods used are not taken into account. Indeed, to target the same element, several methods could exist and could present different PCR efficiencies which could generate variation in the results. Most recently, the JRC-GMO-Matrix platform, combining information from the GMOMETHODS database (all reference methods for GMO analysis) and the Central Core DNA Sequences Information System (several annotated GMO sequences), was also proposed for the same purpose. This platform integrates the positive and negative signals experimentally observed with EU validated taxon-specific, element-specific, construct-specific, and event-specific methods for any tested food/feed matrix in order to predict more reliably the potential amplified GM events [28]. The JRC-GMO-Matrix platform is also strengthened by the JRC GMO-Amplicons database which contains publically available putative GMO-related sequences [132].

#### 2.1.2. Multiplex qPCR Strategy

With multiplex PCR-based methods, several DNA targets can be detected in a single reaction. It presents the advantage to decrease the number of reactions necessary to test the potential presence of GMO in a sample. Several multiplex qPCR TaqMan strategies have thus been investigated, including mainly the screening markers p35S and tNOS (Table 3) [38, 41, 43–49]. To provide a system with a high GMO coverage, twenty-three triplex and one duplex PCR were gathered on a 384-well plate to identify forty-seven targets (Table 3) [42].

However, compared to simplex qPCR, the development of optimal multiplex assays could be more challenging notably in terms of primers and probes design as well as sensitivity and reproducibility. Moreover, the throughput of this strategy is relatively limited by the availability of dyes with an emission and absorption spectrum of fluorescence sufficiently distinct to avoid overlaps of signals. The combination of different dyes risks also increases the fluorescent background. Therefore, the majority of the reported multiplex qPCR assays amplify simultaneously only two or three targets. To date, a maximum of six markers have been successfully combined in one reaction to detect GMO [35, 49].

### 2.2. Alternative Multiplex Strategies

Still with the aim of going further in the development of multiplex assays, several methods not based on qPCR have been also developed using notably the CGE, microarray, and Luminex technologies. Two main steps are generally followed. Firstly, to guarantee a sufficient sensitivity, the samples are amplified by PCR since the GM targets are potentially at trace level in food/feed matrices. In a second step, the PCR products are analyzed using the CGE, microarray, or Luminex platforms. Despite the fact that these technologies present a higher throughput than qPCR, their multiplexing level is still influenced by the inherent properties of PCR which limit the number of reactions at commonly ten targets per PCR assay [133, 134].

#### 2.2.1. PCR Capillary Gel Electrophoresis Technology

In order to detect simultaneously several targets, the use of the PCR multiplex CGE, where fluorescently labelled primers allow discriminating different amplicons of the same size, has been also suggested to be applied in the GMO detection field (Figure 1 and Table 4). Compared to the electrophoresis gel, the resolution power of the CGE system to detect PCR products from a multiplex assay is clearly higher [134]. However, the sensitivity of CGE system is weaker than the qPCR technology [135]. Using the PCR CGE system, eight GM maize were identified via a nonaplex PCR including event-specific, construct-specific, and taxon-specific methods (Table 4) [57, 58]. Similarly, one pentaplex PCR and two hexaplex PCR were also developed to, respectively, detect specifically four GM maize, five GM cotton, and five GM maize (Table 4) [52–55]. Recently, a tetraplex targeting transgenic elements and cotton-specific gene was also reported (Table 4) [51]. In addition, Guo et al., 2011 developed three octaplex PCR using universally tailed primers to preamplify GM targets under a short number of cycles. To increase the yield and PCR efficiency, these amplicons, earlier submitted to a PCR emulsion, are then enriched with universal primers. By this way, twenty-four targets from fourteen GM events were identified by the CGE system (Table 4) [56]. A variant of this technique, which implies no fluorescent labels on primers, is reported by Burrell et al., 2011. This study proposed a tetraplex PCR composed of two species-specific methods and two screening markers allowing detecting the presence of Bt11 maize and GTS40-3-2 soybean events using commercialized electrophoresis instruments (Table 4) [50].

#### 2.2.2. Microarrays Technology

With the microarray technology applied to GMO detection, GM targets are amplified by PCR, using target-specific and/or universal primers, prior to being hybridized on the array, allowing the simultaneous detection of more than 250 000 targets in one assay (Figure 1 and Table 5) [136]. Compared to the qPCR, the microarray strategy presents thus a well higher throughput but a slightly weaker sensitivity [133, 137]. One approach, called multiplex quantitative DNA array-based PCR (MQDA-PCR), tested on transgenic maize events, consists of a first PCR using target-specific primers that harbor a universal tail allowing using universal primers in the second PCR. The signal is then detected after the hybridization of the PCR products with the fluorescently labelled probes on the DNA array (Table 5) [63]. Furthermore, using a padlock probe ligation-microarray detection system (PPLMD), some GM maize, cotton, and soybean events were detected. With the PPLMD system, the targets are initially hybridized to linear padlock probes harboring target-specific and universal sequences to be then amplified by PCR with universal primers (Table 5) [64]. In addition, a nucleic acid sequence based amplification implemented microarray (NAIMA) approach, using universal primers, has been tested on transgenic maize (Table 5) [62, 137]. As an alternative to the potential issue related to the use of fluorescent label, the DualChip GMO system was proposed. So, after PCR amplification with biotinylated target-specific primers, the amplicons hybridized on the arrays are detected by a colorimetric reaction, allowing identifying simultaneously some GM maize, soybean, and rapeseed events. The performance of the DualChip GMO system, targeting fourteen elements, was also validated through an EU collaborative ring trial. An upgraded version of this system (DualChip GMO V2.0) presents a higher GMO coverage in targeting thirty elements (Table 5) [59–61, 133, 138]. Most recently, a multiplex amplification on a chip with readout on an oligo microarray (MACRO) system, targeting ninety-one targets to cover a broad spectrum of GMO, was also reported [139].

#### 2.2.3. Luminex Technology

Biotinylated targets amplified by single or multiplex PCR assays could be analyzed with the Luminex technology, potentially able to simultaneously detect up to 500 different targets in one sample using spectrally distinct sets of beads that are independently coupled to unique nucleic acid probes. After hybridization of biotinylated oligonucleotides to corresponding probe-bead complexes, the reader device individually analyzes each microsphere by flow cytometry in applying a laser excitation of 635 nm and 532 nm allowing, respectively, identifying the bead set and determining the presence or absence of the target (Figure 1) [140]. This technology was firstly assessed in GMO detection by Fantozzi et al., 2008 (Table 6). In this study, the p35S and EPSPS elements, earlier individually amplified by PCR from the GTS-40-3-2 soybean event, were simultaneously detected [65]. Afterwards, the GM stacked LS28 × Cry1Ac rice and 281-24-236 × 3006-210-23 cotton events were identified on the Luminex platform using upstream, respectively, a pentaplex PCR or a hexaplex PCR (Table 6) [67, 68]. This technology was also used to detect ten GM maize events through four sets of multiplex PCR assays (Table 6) [66]. Similarly, a liquid bead array approach allowing identifying thirteen GM maize was recently developed [141].

Due to its potential high throughput, the Luminex technology seems to be a promising alternative in GMO detection. Moreover, the liquid bead array is considered as more sensitive and faster than the microarray system [67]. Nevertheless, the drawback linked to the PCR complicates the setting of a unique multiplex assay targeting simultaneously all GM events. Furthermore, as only few studies using this technology in GMO detection have been reported to date, experiments have still to be carried out in order to provide effective and validated systems.

### 2.3. Digital PCR Technology

To resolve difficulties observed during the relative quantification step in qPCR, especially when the copy numbers of GMO are low and/or PCR inhibitors are present, the digital PCR (dPCR) technology has been tested in GMO detection (Figure 1). Based on the binomial Poisson statistics, each partition of the fractionated sample is determined as positive (amplified target observed) or negative (no amplified target observed) by the dPCR technology allowing absolutely quantifying the number of nucleic acid targets from GMO present in any given sample. Two approaches of this end-point PCR system have up till now been used for this aim (Table 7). On the one hand, the chamber dPCR (cdPCR), partitioning the sample in several thousands of microfluidic chambers, was used to target GM maize MON810 event using a duplex PCR composed of the MON810 event-specific and maize taxon-specific methods. The detection limits of this approach were also investigated [72–74]. Moreover, a strategy based on the cdPCR system was developed in order to cover a wide range of GMO by applying individually twenty-eight element-specific, thirty-six event-specific, and five taxon-specific methods (Table 7) [69]. Afterwards, this strategy was applied with forty-eight markers, including seven transgenic elements-specific, fourteen event-specific, and five taxon-specific methods (Table 7) [70]. On the other hand, the droplet dPCR (ddPCR) approach, implying several thousands of droplets generated by a water-oil emulsion, was used in simplex or duplex PCR with the MON810 event-specific and maize taxon-specific methods [71]. Most recently, duplex assays, including one GMO-specific marker with one soybean, maize, or rice taxon-specific marker, were performed by using the ddPCR system to quantify twelve GM soybean, sixteen GM maize, and two GM rice events (Table 7) [48, 75].

The dPCR technology could become a key tool in the field of GMO detection, mainly because an absolute, and not relative as in qPCR, quantification of the GM target is provided. The measurement does not require necessarily the use of reference material, solving issues related to the availability of an optimal reference material. Moreover, thanks to the partitioning of the sample, the PCR efficiency is less affected by the presence of inhibitors and allows reducing the uncertainty in the measurement, especially at low copy number, as observed with qPCR calibration curves generated by serial dilutions of the target. However, validated qPCR methods are not always simply transferable to the dPCR technology. Indeed, some optimization has to be carried out regarding, for instance, the design and the concentrations of primers and probes. In addition, given that maximum two different targets could be identified in one well, the low throughput power of the dPCR technology highlights its applicability more suitable at the identification/quantification level than at the screening step [48, 71, 75, 142].

### 2.4. Loop-Mediated Isothermal Amplification

Due to its rapidity, specificity, sensitivity, and simplicity, the loop-mediated isothermal amplification (LAMP) method has been proposed to detect GMO (Figure 1). To this end, four primers specific to six distinct regions of the target are required, allowing, under isothermal condition, initiating the reaction and increasing the amplification speed by the formation of a loop structure. The amplification can be then directly visualized in the tube thanks to fluorescent dyes. Several LAMP markers were thus developed for this approach to target transgenic elements (Table 8) [76–91, 143].

The LAMP strategy presents the advantage to tolerate several PCR inhibitors such as acidic polysaccharides [84]. Its implementation does also not require any sophisticate devices. Indeed, the amplification could be carried out using a water bath or heating block [90]. Some of the developed LAMP methods have besides been successfully tested in the fields [84]. Concerning the drawbacks, the design of four primers per target, which guarantee the high specificity and sensitivity of the LAMP, could be difficult. In addition, the identification of several GM targets using a multiplex assay is not applicable [28].

### 2.5. DNA Walking

In using PCR-based methods that required prior knowledge, the observed results are mostly generated in targeting elements derived from natural organisms. Therefore, they constitute merely an indirect proof of the presence of GMO in the tested food/feed matrices. In addition, when the observed signals do not correspond to known GMO, the presence of unknown GMO, containing at least one known element, could be only suspected. The only way to indubitably confirm the presence of GMO is provided by the characterization of sequences from the junctions between the transgenic cassette and the plant genome as well as the unnatural associations of transgenic elements.

To get this crucial information, several strategies of DNA walking, also called genome walking, have been reported (Figure 1 and Table 9). More precisely, this molecular technique allows identifying unknown nucleotide sequences adjacent to already known DNA regions in any given genome using specific primers to the known sequence combined to primers dictated by the DNA walking method used. Then, the final PCR products are usually sequenced by Sanger technology to be eventually analyzed with available databases (e.g., NCBI and JRC GMO-Amplicons). Classically, three main categories of DNA walking are established, based on the characteristics of their first step [144].

First, the restriction-based methods involve a digestion of the genomic DNA using appropriate restriction enzymes targeting sites close to sequences of interest, such as the junction between the known and unknown sequences. The obtained restriction fragments are then either self-circularized or ligated to DNA cassettes, named, respectively, inverted-PCR and cassette PCR methods ([144] and references therein). By this way, several sequences of transgene flanking regions and unnatural associations from transgenic* Arabidopsis thaliana*, tobacco, shallot, potato, barley, grapefruit, tomato, banana, cotton (MON1445), colza (including GT73), soybean (GTS40-3-2 and MON89788), wheat (B73-6-1, B72-8-11, and B72-8-11b), rice (including TC-19, Bt Shanyou 63 (TT51-1), KeFeng-6, and KeFeng-8), and maize (CHB-351, Bt176, GA21, Bt11, MON88017, MON863 × NK603, MON863 × NK603 × MON810, T25, MON810, NK603, MON863, T25, DAS-59122-7, LY038, and 3272) were characterized (Table 9) [92–108, 145–168].

Second, the extension-based methods are defined by the extension of a sequence-specific primer. The resulting single-stranded DNA is subsequently ligated to either a DNA cassette or 3′-tailing ([144] and references therein). This strategy was successfully applied on GM maize (MON810), rice (LLRICE62), soybean (A2704-12), rapeseed (T45), and cotton (LLCOTTON25) events in order to characterize their transgenic cassettes and transgene flanking regions (Table 9) [109, 110].

Third, the primer-based methods combine combinatorial (random and/or degenerate) primers to target-specific primers according to various PCR strategies ([144] and references therein). The transgenic* Arabidopsis thaliana*, tobacco, potato, barley, apple, banana, soybean, wheat (B73-6-1), rice (including KeFeng-6 and KMD1), and maize (including MON863 and MIR162) were thereby identified via the sequences of their transgene flanking regions and unnatural associations of elements (Table 9) [111–116, 152, 154, 157, 169–174].

However, the implementation of most of these DNA walking methods by the enforcement laboratories presents some difficulties such as an insufficient specificity, sensitivity, or yield. Moreover, some of them use laborious, complex, and lengthy techniques (e.g., fingerprinting by capillary electrophoresis and genomic DNA library via (unpredictable) restriction enzyme). Therefore, a DNA walking approach, corresponding better to the need of enforcement laboratories, has been developed and validated on unprocessed and processed food matrices containing minute amounts of GM targets. As this DNA walking approach implies two seminested PCR rounds, the yield and the specificity of GM targets are increased, especially crucial in case of a low level presence of GMO. This approach, belonging to the PCR-based method category, has also the advantage to be fully integrated into the GMO routine analysis as the similar primers are used for the qPCR screening (detection of potential GMO presence) and the DNA walking (GMO identification). So, this simple and rapid approach could easily be applied by the enforcement laboratories, without any significant additional cost and equipment, to confirm signals previously obtained in qPCR (Table 9) [33, 117, 118].

Since DNA walking requires less prior knowledge about the sequence of interest than conventional PCR-based methods previously described, GMO with entirely or partially known sequences could be characterized. Therefore, in targeting key elements, such as p35S and tNOS that are highly frequent in GM crops, a broad range of GMO could be characterized [96, 106, 110, 111, 113, 118, 156]. In order to especially identify unauthorized GMO in European Union, a DNA walking approach using primers specific to the element t35S from the pCAMBIA vector, found in approximately 30% of transgenic plants, was developed [33, 117]. However, the DNA walking strategy is not suitable to GMO containing only unknown elements.

### 2.6. Next Generation Sequencing Technologies

Despite their higher throughput compared to qPCR, the multiplex strategies described above require the prior knowledge of at least a part of the GMO sequences. Once the information about these sequences is collected, the development of methods, each one targeting indivdually one sequence of interest, is carried out on a case-by-case basis. Then, the optimisation of unbiased multiplex assays presenting equal analytical performance compared to simplex assays remains laborious and intricate. Furthermore, the issues related to the detection of GMO containing no known sequences are still unsolved. Recently, NGS, allowing a massive parallel DNA sequencing, has been suggested to tackle these challenges. The NGS technology outperforms plainly the classical Sanger sequencing in terms of rapidity and throughput. Indeed, the powerful high throughput of NGS offers the possibility to sequence simultaneously many different samples, discriminable in using a wide range of barcodes [116, 124, 175]. Two main strategies, sequencing samples that are earlier enriched with sequences of interest (targeted sequencing approach) or not (whole genome sequencing (WGS) approach), exist (Figure 1 and Table 10).

#### 2.6.1. Targeted Sequencing

The targeted sequencing strategy is especially beneficial to target regions of interest from large and complex genomes, observed in most of plants. Even if a minimum of prior knowledge on sequences is needed to target the sequences of interest, it presents the advantage to use exclusively all the energy, in terms of time and cost, on the regions of interest. With this strategy, two substrategies could be used, involving the sequencing of either DNA library of PCR products (amplicon sequencing) or selected DNA fragments from a whole genome library (target enrichment sequencing) (Figure 1).

On the one hand, as the amplicon sequencing allows characterizing DNA fragments of interest previously enriched by PCR, this sequencing approach depends thus clearly on the PCR strategy adopted upstream as well as its inherent properties and performance. In order to detect GMO, Song et al., 2014 generated amplicons by PCR, using primers targeting maize endogen gene, Bt11 gene, Bt176 gene, soybean endogen gene, 35S/CTP4 construct, CP4-EPSPS element, p35S promoter, and tNOS terminator, from samples containing a low amount of GM targets (1% of Bt11 maize, 2% of Bt176 maize, 2% of GTS40-3-2 soybean, 1% of GTS40-3-2 soybean, 0.1% of GTS40-3-2 soybean, or 0.01% of GTS40-3-2 soybean). Then, each kind of amplicons was individually sequenced using a variant of the 454 system called pyrosequencing on portable photodiode-based bioluminescence sequencer that is more sensitive, compact, and cost-efficient compared to the original 454 technology (Roche) (Table 10) [119, 176]. This approach is relatively similar to the PCR screening with the additional value to provide, instead of positive or negative signals, the sequence of the amplified fragments, which is more reliable to prove the presence of GMO. Conversely to this approach, Liang et al., 2014 suggest an amplicon sequencing strategy allowing analyzing GMO for which the sequence information is only partially known. To this end, a DNA walking method (SiteFinding PCR), targeting the vip3Aa20 sequence, was coupled to NGS technologies, using the Illumina or Pacific Biosciences platforms, to characterize the sequences of the MIR162 maize event (Table 10). Even if the results were similar using the two different NGS platforms, the PacBio system shows the advantage to sequence DNA fragments with a size reaching up to 40 Kbp and to deal with DNA fragments presenting different sizes. Therefore, the PacBio system, in contrast to the Illumina technology, allows in many cases avoiding a de novo assembly step as the shearing of genomic DNA is not always required. Moreover, the use of NGS instead of the Sanger technology allows considerably increasing the throughput of DNA walking approaches. Indeed, in order to guarantee the entire representativeness of GMO present in a tested sample, all observed amplicons should be analyzed. However, the purification of the potential numerous amplicons excised from the electrophoresis gel and the subsequent Sanger sequencing could be cumbersome, especially in case of food/feed matrices containing several GMO sharing common targeted elements [116, 118, 177].

On the other hand, the target enrichment sequencing approach involves the selection of sequences of interest from the whole genome DNA library. To capture them, appropriate hybridization methods could be used relying on magnetic beads or microarrays associated with specific probes. The efficiency of the hybridization step is thus crucial for this sequencing strategy. The DNA fragments containing entirely or partially the known regions could be then sequenced. However, even if this strategy has been applied to different plants, no study has to date been reported to our knowledge to detect GMO [178–181].

The analysis of preenriched DNA fragments of interest with NGS technology allows proving the presence of GMO in characterizing sequences entirely or partially known beforehand. However, given its relative high cost, expected to decrease over the time, and the prerequisite bioinformatics expertise, the targeted NGS strategy could not reasonably be currently applied routinely to all food/feed matrices by the enforcement laboratories [116, 124, 175].

#### 2.6.2. Whole Genome Sequencing

The WGS strategy allows in principle characterizing a sample without any prior knowledge (Figure 1). With this sequencing strategy, the entire DNA library, consisting of sheared genomic DNA ligated to adaptors, is sequenced. The generated reads are then treated with bioinformatics tools based on prior knowledge of tested GMO.

First, when no information about the transgenic cassette is available, the insert and its transgene flanking regions are identified by the analysis of all inferred contigs derived from reads that partially matched or unmatched with the endogenous plant-species reference genome [123]. This WGS strategy was applied on the LLRICE62 event by using the available reference genome of* Oryza sativa *ssp. Japonica. As the results corresponded to the information from the developer dossier, the characterization of GMO with an unknown insert using NGS was thus demonstrated (Table 10) [122]. Similarly, the T-DNA regions from the GM flax FP967 event and the transgenic rice TT51-1 and T1c-19 events were also characterized (Table 10) [121, 123]. The success of this strategy is thus linked to the availability of good reference genomes for specific varieties and organisms. In case of no reference genome available, a strategy of de novo assembly, comparing all generated reads to find overlaps, has to be applied. However, this remains quite cumbersome with the large and complex plant genomes notably in terms of ploidy, repeated regions, and heterozygosity and with mixtures of different GMO [120, 182]. To facilitate even so the de novo assembly, the strength of different NGS platforms can be associated. For instance, short reads from Illumina technology can be aligned to long reads generated by the PacBio technology, constituting a substitute of reference genome [183].

Second, with the condition that the sequence of at least one transgenic element is known, the insert is de novo assembled with reads that are matched and unmatched with a DNA transgene sequence library containing frequently used transgenic elements. This approach was tested on the transgenic rice TT51-1 and T1c-19 events (Table 10) [123].

Third, if the sequence of the insert is known, two kinds of bioinformatics analysis have been reported. On the one hand, the reads, corresponding not entirely to the reference genome, are mapped to the transgenic cassette sequence in order to determine the number of inserts and their transgene flanking regions. By this way, the GM rice TT51-1 and T1c-19 events and the GM soybean MON17903 and MON87704 events were characterized (Table 10) [120, 123]. On the other hand, Willems et al., 2016 have developed an analytical workflow, including three different approaches. The detection approach, consisting of comparing the reads to the reference sequence of the insert, allows detecting the presence of GMO in a given sample. To confirm the integration of the transgenic cassette and provide a rough localization of its flanking regions, the matched reads are then compared to the reference sequence of the host genome in the proof approach. By the simultaneous aligning of these selected reads to the host genome and the transgenic cassette, the identification approach allows determining precisely the localization of the transgenic cassette and the sequence of its flanking regions. This WGS strategy was initially assessed on pure transgenic GM rice (100% Bt rice). Conversely to all the other WGS strategies described above, food/feed matrices more likely to be encountered in GMO routine analysis, such as a GM/non-GM rice mixture (10% Bt rice) and a processed GM rice (100% Bt noodles), have also been tested (Table 10) [124]. In this study, a statistical framework, predicting the probability to detect a sequence derived from a transgenic cassette and validated with experimental data originated from WGS, was also developed to estimate* in silico* the number of reads, derived from Illumina HiSeq device, required to characterize frequently encountered GMO. It was shown that samples composed of GMO at 100%, except for GM wheat owning a huge genome, could be wisely characterized at a standard price range. A contrario, the detection, and identification of GMO present at trace level are not reasonably achievable by WGS [124]. Therefore, at the present time, only the previously described targeted sequencing approach can be applied on GM mixture containing GMO at trace level within reason.

The NGS technology is thus a promising alternative in the GMO detection field which offers the possibility to prove straightforward the presence of GMO in food/feed matrix via the characterization of their sequences. Moreover, the sequences obtained from unknown GMO will allow designing new PCR markers. Nevertheless, the implementation of NGS in GMO routine analysis by the enforcement laboratories is still difficult due to its relatively high cost as well as the requirement of adequate computer infrastructures and qualified analysts in bioinformatics for dealing with the generated data [116, 124, 175].

## 3. Conclusion

In GMO routine analysis, qPCR remains the method of choice for the enforcement laboratories. However, as some technical hurdles could be encountered with this technology, alternative GMO detection methods have been developed to raise some of these challenges. In order to exploit at best the performance of all the above described strategies, their applicability could be considered according to the adopted strategy of GMO detection as well as the available information about the sequences of tested GMO (Figure 1). In case of fully characterized GMO, the methods based on conventional PCR are absolutely appropriate to rapidly detect individually GM targets low-prized (LAMP), to simultaneously detect several GM targets (CGE, microarray, and Luminex) or to precisely quantify the amount of GM targets without impact of inhibitors (dPCR). However, when tested matrices contain GMO for which only a part of their sequences is known, these strategies could generate unexplained signals for which the observed positive signals could not be related to known GM events. In targeting key DNA sequences, such as the elements p35S and tNOS that are frequently found in GM plants, the use of DNA walking or targeted sequencing by enrichment strategies allows indubitably confirming the presence of GMO via the sequences of transgenes flanking regions and unnatural associations of genetic elements. If no information is available, at this moment, only the WGS is conceivable to characterize this category of GMO.

## Figures and Tables

**Table 1:** Representative examples illustrating protein-based methods targeting GMO.

Technologies	Targets	References
ELISA	CP4-EPSPS	[4]
	Cry1Ab	[10, 12, 15, 18, 20]
	Cry1Ac	[10, 14]
	Cry2A	[10]
	Cry2Ab	[19]
	Cry3A	[10, 16]
	Cry9C	[10]
	nptII	[5, 16, 22]
	CP4-EPSPS	[6, 10, 13, 22]
	pat	[10, 11, 13, 22]
	Gox	[17]
	CpTI	[21]

Immuno-PCR	Cry1Ac	[23]
	p35S	[24]
	tNOS	[24]

CP4-EPSPS (5-enolpyruvylshikimate-3-phosphate synthase gene from *Agrobacterium tumefaciens *strain); CpTI (trypsin inhibitor in cowpea *Vigna unguiculata*); Cry (gene encoding the *Bacillus thuringiensis δ*-endotoxin); Gox (glyphosate oxidoreductase gene); nptII (neomycin phosphotransferase II gene); p35S (promoter of the 35 S cauliflower mosaic virus); tNOS (terminator of the nopaline synthase gene).

**Table 2:** Representative examples illustrating simplex qPCR methods targeting GMO. Those validated at the EU level are indicated by an asterisk. Screening markers used in the CoSYPS are indicated by ~.

Methods	Chemistries	Targets		References
Screening markers				

Plant-specific	SYBR Green	RBCL^~^		[37]
Taxon-specific	SYBR Green	LEC^*∗*~^		[35]
	SYBR Green	ADH^*∗*~^		[37]
	SYBR Green	CRU^*∗*~^		[37]
	SYBR Green	PLD^~^		[37]
	SYBR Green	SAD1^~^		[35]
	SYBR Green	GLU^~^		[35]
Element-specific	SYBR Green	p35S^*∗*~^		[31]
	TaqMan	p35S^*∗*^		[38]
	SYBR Green	tNOS^*∗*~^		[31]
	TaqMan	tNOS^*∗*^		[38]
	SYBR Green	pFMV^~^		[39]
	TaqMan	pFMV^*∗*^		[35]
	SYBR Green	pNOS^~^		[39]
	SYBR Green	t35S^~^		In-house
	SYBR Green	Cry1Ab/Ac^~^		[40]
	TaqMan	Cry1A(b)^*∗*^		[35]
	SYBR Green	Cry3Bb^~^		[34]
	SYBR Green	pat^*∗*~^		[40]
	TaqMan	pat^*∗*^		[35]
	SYBR Green	bar^*∗*~^		[40]
	TaqMan	bar^*∗*^		[35]
	SYBR Green	CP4-EPSPS^~^		[40]
	SYBR Green	t35S pCAMBIA^~^		[33]
	SYBR Green	nptII		[35]
Construct-specific	SYBR Green	gat-tpinII^~^		[34]
Virus-specific	SYBR Green	CRT^~^		In-house

Event-specific methods				

GM-specific	TaqMan	Maize (*Zea mays*)	3272^*∗*^	[35]
	TaqMan	Maize (*Zea mays*)	5307^*∗*^	[35]
	TaqMan	Maize (*Zea mays*)	98140^*∗*^	[35]
	TaqMan	Maize (*Zea mays*)	Bt11^*∗*^	[35]
	TaqMan	Maize (*Zea mays*)	Bt176^*∗*^	[35]
	TaqMan	Maize (*Zea mays*)	DAS-40278-9^*∗*^	[35]
	TaqMan	Maize (*Zea mays*)	DAS-59122-7^*∗*^	[35]
	TaqMan	Maize (*Zea mays*)	GA21^*∗*^	[35]
	TaqMan	Maize (*Zea mays*)	LY038^*∗*^	[35]
	TaqMan	Maize (*Zea mays*)	MIR162^*∗*^	[35]
	TaqMan	Maize (*Zea mays*)	MIR604^*∗*^	[35]
	TaqMan	Maize (*Zea mays*)	MON810^*∗*^	[35]
	TaqMan	Maize (*Zea mays*)	MON863^*∗*^	[35]
	TaqMan	Maize (*Zea mays*)	MON87460^*∗*^	[35]
	TaqMan	Maize (*Zea mays*)	MON88017^*∗*^	[35]
	TaqMan	Maize (*Zea mays*)	MON89034^*∗*^	[35]
	TaqMan	Maize (*Zea mays*)	NK603^*∗*^	[35]
	TaqMan	Maize (*Zea mays*)	T25^*∗*^	[35]
	TaqMan	Maize (*Zea mays*)	TC1507^*∗*^	[35]
	TaqMan	Soybean (*Glycine max*)	A2704-12^*∗*^	[35]
	TaqMan	Soybean (*Glycine max*)	A5547-127^*∗*^	[35]
	TaqMan	Soybean (*Glycine max*)	BPS-CV-127^*∗*^	[35]
	TaqMan	Soybean (*Glycine max*)	DAS68416-4^*∗*^	[35]
	TaqMan	Soybean (*Glycine max*)	DP-305423-1^*∗*^	[35]
	TaqMan	Soybean (*Glycine max*)	DP-356043-5^*∗*^	[35]
	TaqMan	Soybean (*Glycine max*)	FG72^*∗*^	[35]
	TaqMan	Soybean (*Glycine max*)	GTS40-3-2^*∗*^	[35]
	TaqMan	Soybean (*Glycine max*)	MON87701^*∗*^	[35]
	TaqMan	Soybean (*Glycine max*)	MON87705^*∗*^	[35]
	TaqMan	Soybean (*Glycine max*)	MON87708^*∗*^	[35]
	TaqMan	Soybean (*Glycine max*)	MON87769^*∗*^	[35]
	TaqMan	Soybean (*Glycine max*)	MON89788^*∗*^	[35]
	TaqMan	Cotton (*Gossypium hirsutum*)	281-24-236^*∗*^	[35]
	TaqMan	Cotton (*Gossypium hirsutum*)	3006-210-23^*∗*^	[35]
	TaqMan	Cotton (*Gossypium hirsutum*)	GHB119^*∗*^	[35]
	TaqMan	Cotton (*Gossypium hirsutum*)	GHB614^*∗*^	[35]
	TaqMan	Cotton (*Gossypium hirsutum*)	LLCOTTON25^*∗*^	[35]
	TaqMan	Cotton (*Gossypium hirsutum*)	MON531^*∗*^	[35]
	TaqMan	Cotton (*Gossypium hirsutum*)	MON1445^*∗*^	[35]
	TaqMan	Cotton (*Gossypium hirsutum*)	MON15985^*∗*^	[35]
	TaqMan	Cotton (*Gossypium hirsutum*)	MON88913^*∗*^	[35]
	TaqMan	Cotton (*Gossypium hirsutum*)	T304-40^*∗*^	[35]
	TaqMan	Oilseed rape (*Brassica napus*)	73496^*∗*^	[35]
	TaqMan	Oilseed rape (*Brassica napus*)	GT73^*∗*^	[35]
	TaqMan	Oilseed rape (*Brassica napus*)	MON88302^*∗*^	[35]
	TaqMan	Oilseed rape (*Brassica napus*)	Ms1^*∗*^	[35]
	TaqMan	Oilseed rape (*Brassica napus*)	Ms8^*∗*^	[35]
	TaqMan	Oilseed rape (*Brassica napus*)	Rf1^*∗*^	[35]
	TaqMan	Oilseed rape (*Brassica napus*)	Rf2^*∗*^	[35]
	TaqMan	Oilseed rape (*Brassica napus*)	Rf3^*∗*^	[35]
	TaqMan	Oilseed rape (*Brassica napus*)	T45^*∗*^	[35]
	TaqMan	Oilseed rape (*Brassica napus*)	Topas 19/2^*∗*^	[35]
	TaqMan	Potato (*Solanum tuberosum*)	EH92-527-1^*∗*^	[35]
	TaqMan	Rice (*Oryza sativa*)	LLRICE62^*∗*^	[35]
	TaqMan	Sugar beet (*Beta vulgaris*)	H7-1^*∗*^	[35]

ADH (alcohol dehydrogenase I gene from maize); bar (phosphinothricin-N-acetyltransferases gene from *Streptomyces hygroscopicus*); CP4-EPSPS (5-enolpyruvylshikimate-3-phosphate synthase gene from *Agrobacterium tumefaciens* strain); CRT (reverse transcriptase gene from the cauliflower mosaic virus); CRU (cruciferin gene from colza); Cry (gene encoding the *Bacillus thuringiensis δ*-endotoxin); gat-tpinII (junction sequence between the glyphosate N-acetyltransferase of *Bacillus licheniformis* and the terminator of the *Solanum tuberosum* proteinase inhibitor); GLU (glutamine synthetase gene from sugar beet); LEC (lectin gene from soybean); nptII (neomycin phosphotransferase II gene); p35S (promoter of the 35 S cauliflower mosaic virus); pat (phosphinothricin-N-acetyltransferases gene from *Streptomyces viridochromogenes*); pFMV (promoter of the figwort mosaic virus); phy (phytase gene from maize); PLD (phospholipase D gene from rice); pNOS (promoter of the nopaline synthase gene); RBCL (ribulose-1,5-biphosphate carboxylase oxygenase); SAD1 (stearoyl-acyl carrier protein desaturase gene from cotton); t35S (terminator of the cauliflower mosaic virus); t35S pCAMBIA (terminator of the cauliflower mosaic virus from pCAMBIA vector); tNOS (terminator of the nopaline synthase gene).

**Table 3:** Representative examples illustrating multiplex qPCR TaqMan methods targeting GMO. Those validated at the EU level are indicated by an asterisk.

Multiplexing	Methods	Targets	References
Duplex	Element-specific	p35S^*∗*^ and tNOS^*∗*^	[38]
Duplex	Element-specific	bar and pat	[41]
Duplex	Plant-specific	TLC	[42]
	Other	IPC	
Duplex	Taxon-specific	ADH	[43]
	Event-specific	Bt11	
Duplex	Taxon-specific	ADH	[43]
	Event-specific	Bt176	
Duplex	Taxon-specific	ADH	[43]
	Event-specific	MON810	
Duplex	Taxon-specific	ADH	[43]
	Event-specific	T25	
Triplex	Element-specific	p35S, tNOS, and CTP2/CP4-EPSPS	[41]
Triplex	Taxon-specific	LEC and Zein	[42]
	Other	IPC	
Triplex	Taxon-specific	Pro and PC	[42]
	Other	IPC	
Triplex	Taxon-specific	ACC and FRUp	[42]
	Other	IPC	
Triplex	Taxon-specific	SAD1 and FRUt	[42]
	Other	IPC	
Triplex	Element-specific	p35S and pFMV	[42]
	Other	IPC	
Triplex	Element-specific	tE9 and tNOS	[42]
	Other	IPC	
Triplex	Element-specific	bar and CP4-EPSPS	[42]
	Other	IPC	
Triplex	Element-specific	hpt and pat	[42]
	Other	IPC	
Triplex	Element-specific	nptII and Cry1Ab/Ac	[42]
	Other	IPC	
Triplex	Construct-specific	CBH351 and Bt176	[42]
	Other	IPC	
Triplex	Construct-specific	MON810 and T25	[42]
	Other	IPC	
Triplex	Construct-specific	Bt11 and MON863	[42]
	Other	IPC	
Triplex	Construct-specific	NK603 and GA21	[42]
	Other	IPC	
Triplex	Construct-specific	TC1507 and DAS-59122-7	[42]
	Other	IPC	
Triplex	Construct-specific	MIR604 and MON88017	[42]
	Other	IPC	
Triplex	Construct-specific	98140 and MON89034	[42]
	Other	IPC	
Triplex	Construct-specific	3272 and MIR162	[42]
	Other	IPC	
Triplex	Construct-specific	A2704-12 and GTS40-3-2	[42]
	Other	IPC	
Triplex	Construct-specific	DP-305423-1 and DP-356043-5	[42]
	Other	IPC	
Triplex	Construct-specific	MON87701 and MON89788	[42]
	Other	IPC	
Triplex	Element-specific	AHAS	[42]
	Construct-specific	FG72	
	Other	IPC	[42]
Triplex	Construct-specific	Bt63 and A5547-127	[42]
	Other	IPC	
Triplex	Element-specific	Xa21	
	Construct-specific	KMD1	
	Other	IPC	
Triplex	Taxon-specific	Zein	[44]
	Construct-specific	MON810 and GA21	
Triplex	Taxon-specific	ADH	[44]
	Construct-specific	MON810 and GA21	
Triplex	Element-specific	p35s, tNOS, and t35S	[45]
Triplex	Element-specific	tE9, pRbcS4, and tORF23	[45]
Triplex	Element-specific	tpinII and tAHASL	[45]
	Event-specific	DP-305423-1	
Tetraplex	Element-specific	pFMV, bar, pat, and CTP2/CP4-EPSPS	[46]
Tetraplex	Element-specific	p35S, tNOS, pFMV, and bar	[47]
Pentaplex	Taxon-specific	HMG and LEC	
	Element-specific	p35S and tNOS	[46]
	Virus-specific	CaMV	
Pentaplex	Element-specific	p35S, tNOS, bar, pat, and CTP2/CP4-EPSPS	[41]
Pentaplex	Taxon-specific	LEC	[48]
	Event-specific	MON87769, MON87708, MON87705, and FG72	
Hexaplex	Element-specific	p35S, tNOS, and pFMV	
	Construct-specific	SAMS and LY	[49]
	Other	IPC	

ACC (acetyl-CoA-carboxylase gene from colza); ADH (alcohol dehydrogenase I gene from maize); AHAS (AHAS fragment unique recombination from BPS-CV-127); bar (phosphinothricin-N-acetyltransferases gene from *Streptomyces hygroscopicus*); CaMV (ORFIII from CaMV); CP4-EPSPS (5-enolpyruvylshikimate-3-phosphate synthase gene from *Agrobacterium tumefaciens* strain); Cry (gene encoding the *Bacillus thuringiensis δ*-endotoxin); CTP2/CP4-EPSP (junction region between the chloroplast transit peptide 2 (CTP2) sequence from the *Arabidopsis thaliana* epsps gene and the CP4 epsps gene from *Agrobacterium tumefaciens* (CP4-EPSPS)); FRUp (*β*-fructosidase gene from potato); FRUt (*β*-fructosidase gene from tomato); HMG (major high-mobility group protein gene from maize); hpt (hygromycin phosphotransferase gene); IPC (internal positive control); LEC (lectin gene from soybean); LS28 (choline kinase); LY (transition from *Zea may*s chloroplast transit peptide sequence for dihydrodipicolinate synthase to *Corynebacterium glutamicum* dihydrodipicolinate synthase (cordapA) gene encoding for a lysine-insensitive dihydrodipicolinate synthase enzyme); nptII (neomycin phosphotransferase II gene); p35S (promoter of the 35 S cauliflower mosaic virus); pat (phosphinothricin-N-acetyltransferases gene from *Streptomyces viridochromogenes*); PC (phosphoenolpyruvate carboxylase gene from wheat); pFMV (promoter of the figwort mosaic virus); pRbcS4 (ribulose 1,5-bisphosphate carboxylase small subunit promoter from *A. thaliana*); Pro (prolamin gene from rice); SAD1 (stearoyl-acyl carrier protein desaturase gene from cotton); SAMS (transition from S-adenosyl-L-methionine synthetase (SAMS) promoter to *Glycine max* acetolactate synthase (gm-hra) gene); t35S (terminator of the cauliflower mosaic virus); tAHASL (acetohydroxy acid synthase large subunit terminator from *A. thaliana*); tE9 (ribulose-1,5-bisphosphate carboxylase terminator E9 from *Pisum sativum*); TLC (tRNA-Leu chloroplastic gene); tNOS (terminator of the nopaline synthase gene); tORF23 (open reading frame 23 terminator from *A. tumefaciens*); tpinII (inhibitor II terminator from potato); Zein (Zein gene from maize), Xa21 (Xa21 gene from *Oryza longistaminata*).

**Table 4:** Representative examples illustrating multiplex PCR CGE methods targeting GMO.

Multiplexing	Methods	Targets	References
Tetraplex	Taxon-specific	Zein and LEC	[50]
	Element-specific	p35S and tNOS	
Tetraplex	Taxon-specific	SAD1	[51]
	Element-specific	Cry1Ac, p35S, and tNOS	
Pentaplex	Taxon-specific	ADH	[52]
	Event-specific	Bt11, GA21, MON810, and NK603	
Hexaplex	Taxon-specific	acp1	[53]
	Event-specific	Bollgard, Bollgard II, RR, 3006-210-23, and 281-24-231	
Hexaplex	Taxon-specific	HMG	[54, 55]
	Event-specific	DAS-59122-7, LY038, MON88017, MIR604, and 3272	
Octaplex	Event-specific	Bt11, Bt176, Huanong No. 1, GTS40-3-2, T25, MON88913, MON1445, and MIR604	[56]
Octaplex	Taxon-specific	LEC and ssIIb	
	Element-specific	pFMV and tNOS	[56]
	Event-specific	TC1507, MON531, NK603, and GA21	
Octaplex	Taxon-specific	SAD1	
	Element-specific	bar, chy, pAct, CP4-EPSPS, and Cry1Ab	[56]
	Event-specific	GT73 and OXY235	
Nonaplex	Taxon-specific	HMG	[57, 58]
	Event-specific	T25, GA21, TC1507, MON863, MON810, NK603, Bt176, and Bt11	

acp1 (acyl carrier protein 1 gene from cotton); ADH (alcohol dehydrogenase I gene from maize); bar (phosphinothricin-N-acetyltransferases gene from *Streptomyces hygroscopicus*); Chy (chymopapain gene from papaya); CP4-EPSPS (5-enolpyruvylshikimate-3-phosphate synthase gene from *Agrobacterium tumefaciens* strain); Cry (gene encoding the *Bacillus thuringiensis δ*-endotoxin); HMG (major high-mobility group protein gene from maize); LEC (lectin gene from soybean); p35S (promoter of the 35 S cauliflower mosaic virus); pAct (promoter region of rice actin gene); pFMV (promoter of the figwort mosaic virus); SAD1 (stearoyl-acyl carrier protein desaturase gene from cotton); ssIIb (starch synthase IIb gene from maize); tNOS (terminator of the nopaline synthase gene); Zein (Zein gene from maize).

**Table 5:** Representative examples illustrating multiplex PCR microarray methods targeting GMO.

Multiplexing	Techniques	Methods	Targets	References
Duplex	DualChip GMO	Element-specific	p35S and tNOS	[59–61]
Duplex	DualChip GMO	Construct-specific	pNOS/nptII	[59–61]
		Virus-specific	CaMV	
Triplex	DualChip GMO	Element-specific	pat, Cry1A(b), and CP4-EPSPS	[59–61]
Triplex	NAIMA	Taxon-specific	IVR	[62]
		Element-specific	p35S and tNOS	
Triplex	NAIMA	Taxon-specific	IVR	
		Element-specific	p35S	[62]
		Event-specific	MON810	
Tetraplex	DualChip GMO	Plant-specific	RBCL	[59–61]
		Taxon-specific	IVR, LEC, and CRU	[63]
Octaplex	MQDA-PCR	Taxon-specific	HMG	
		Element-specific	p35S and tNOS	
		Event-specific	Bt176, Bt11, and MON810	
		Other	IPC	
Decaplex	PPLMD	Taxon-specific	SAD1, Zein, ACC, and LEC	
		Element-specific	p35S, pFMV, and bar	[64]
		Event-specific	MON1445, Bt176, and GTS40-3-2	
Dodecaplex	MQDA-PCR	Taxon-specific	HMG	[63]
		Element-specific	p35S, tNOS, and Amp	
		Event-specific	Bt176, Bt11, MON810, T25, GA21, CBH351, and DBT418	
		Other	IPC	

ACC (acetyl-CoA-carboxylase gene from colza); Amp (ampicillin resistance gene); bar (phosphinothricin-N-acetyltransferases gene from *Streptomyces hygroscopicus*); CaMV (ORFIII from CaMV); CP4-EPSPS (5-enolpyruvylshikimate-3-phosphate synthase gene from *Agrobacterium tumefaciens* strain); CRU (cruciferin gene from colza); Cry (gene encoding the *Bacillus thuringiensis δ*-endotoxin); HMG (major high-mobility group protein gene from maize); IPC (internal positive control); IVR (invertase gene from maize); LEC (lectin gene from soybean); nptII (neomycin phosphotransferase II gene); p35S (promoter of the 35 S cauliflower mosaic virus); pat (phosphinothricin-N-acetyltransferases gene from *Streptomyces viridochromogenes*); pFMV (promoter of the figwort mosaic virus); pNOS (promoter of the nopaline synthase gene); RBCL (ribulose-1,5-biphosphate carboxylase oxygenase); SAD1 (stearoyl-acyl carrier protein desaturase gene from cotton); tNOS (terminator of the nopaline synthase gene); Zein (Zein gene from maize).

**Table 6:** Representative examples illustrating Luminex strategies targeting GMO.

Multiplexing	Methods	Targets	References
Simplex	Element-specific	p35S and CP4-EPSPS	[65]
Triplex	Taxon-specific	Zein	[66]
	Event-specific	MIR604 and MON88017	
Tetraplex	Event-specific	Bt176, MON810, NK603, and GA21	[66]
Tetraplex	Event-specific	Bt11, T25, MIR162, and MON89034	[66]
Pentaplex	Taxon-specific	SPS	[67]
	Element-specific	Cry1Ac, tNOS, p35S, and LS28	
Hexaplex	Taxon-specific	SAD1	
	Element-specific	Cry1Ac, Cry1F, and pat	[68]
	Event-specific	281-24-236 and 3006-210-23	

CP4-EPSPS (5-enolpyruvylshikimate-3-phosphate synthase gene from *Agrobacterium tumefaciens* strain); Cry (gene encoding the *Bacillus thuringiensis δ*-endotoxin); LS28 (choline kinase); p35S (promoter of the 35 S cauliflower mosaic virus) SAD1 (stearoyl-acyl carrier protein desaturase gene from cotton); SPS (sucrose phosphate synthase gene from rice); tNOS (terminator of the nopaline synthase gene); tORF23 (open reading frame 23 terminator from *A. tumefaciens*); Zein (Zein gene from maize).

**Table 7:** Representative examples illustrating digital PCR strategies targeting GMO.

Multiplexing	Techniques	Methods	Targets	References
Simplex	cdPCR	Taxon-specific	HMG, LEC, GLU, and CRU	[69]
		Element-specific	p35S, tNOS, Cry1Ab, Cry1F, bar, CP4-EPSPS, Cry3Bb, nptII, Cry1A.105, and Cry2Bb	
		Event-specific	MON531, MON88913, MON1445, MON15985, LLCOTTON25, GHB614, 3272, DAS-59122-7, Bt176, Bt11, GA21, MIR162, MIR604, MON810, MON863, MON88017, MON89034, NK603, T25, TC1507, Ms1, Topas19/2, OXY_235, Ms8, Rf3, GT73, T45, GTS40-3-2, A2704-12, MON89788, MON87701, DP-356043-5, A5547-127, BPS-CV-127, DP-305423-1, and TT51-1	

Simplex	cdPCR	Taxon-specific	ADH, CRU, PLD, LEC, and adhC	[70]
		Element-specific	p35S, pFMV, tNOS, Cry1Ab, bar, pat, and nptII	
		Event-specific	3272, Bt11, GA21, MON89034, MON810, MIR604, MON88017, TC1507, Bt176, GTS40-3-1, DP-305423-1, DP-356043-5, H7-1, and GT73	

Simplex	ddPCR	Taxon-specific	HMG	[71]
		Event-specific	MON810	

Duplex	cdPCR	Taxon-specific	HMG	[72–74]
		Event-specific	MON810	

Duplex	ddPCR	Taxon-specific	LEC	[48, 75]
		Event-specific	DP-356043-5, DP-305423-1, MON89788, GTS40-3-2, A5547-127, BPS-CV-127, A2704-12, MON87701, MON87708, MON87705, FG72, and MON87769	

Duplex	ddPCR	Taxon-specific	PLD	[75]
		Event-specific	LLRICE62 and KMD1	

Duplex	ddPCR	Taxon-specific	HMG	[75]
		Event-specific	Bt176, Bt11, MON810, NK603, Starllink, MON863, GA21, DAS-59122-7, MIR162, MIR604, 3272, T25, TC1507, MON88017, MON89034, and DAS-40278-9	

Duplex	ddPCR	Taxon-specific	HMG	[71]
		Event-specific	MON810	

ADH (alcohol dehydrogenase I gene from maize); adhC (alcohol dehydrogenase C gene from cotton); bar (phosphinothricin-N-acetyltransferases gene from *Streptomyces hygroscopicus*); CP4-EPSPS (5-enolpyruvylshikimate-3-phosphate synthase gene from *Agrobacterium tumefaciens* strain); CRU (cruciferin gene from colza); Cry (gene encoding the *Bacillus thuringiensis δ*-endotoxin); GLU (glutamine synthetase gene from sugar beet); HMG (major high-mobility group protein gene from maize); LEC (lectin gene from soybean); nptII (neomycin phosphotransferase II gene); p35S (promoter of the 35 S cauliflower mosaic virus); pat (phosphinothricin-N-acetyltransferases gene from *Streptomyces viridochromogenes*); pFMV (promoter of the figwort mosaic virus); phy (phytase gene from maize); PLD (phospholipase D gene from rice); pNOS (promoter of the nopaline synthase gene); tNOS (terminator of the nopaline synthase gene).

**Table 8:** Representative examples illustrating simplex LAMP strategies targeting GMO.

Methods	Targets	References
Taxon-specific	ADH	[76]
	LEC	[77, 78]
	PLD	[79]
	IVR	[80]

Element-specific	p35S	[76, 81–86]
	pFMV	[83, 86]
	aadA	[83]
	uidA	[83]
	nptII	[83, 86]
	Cry1Ab	[87]
	tNOS	[76, 78, 82, 84, 86]
	pNOS	[82]
	bar	[84, 86]
	pat	[86]
	Cry1Ac	[86]
	CP4-EPSPS	[86]
	Cry2A	[88]
	Cry3A	[88]
	phy	[89]

Construct-specific	p35S/EPSPS	[82]

Event-specific	Ms8	[82]
	Rf3	[82]
	MON89788	[77, 78, 84]
	GTS 40-3-2	[77, 78, 84]
	DAS-59122-7	[80, 84]
	MON863	[80, 84]
	TC1507	[80, 84]
	T25	[80, 90]
	Bt11	[80]
	Bt176	[80]
	MON810	[80]
	B73-6-1	[91]
	KMD1	[79]
	Kefeng-6	[79]
	TT51-1	[79]

aadA (aminoglycoside 3′-adenylyltransferase); ACC (acetyl-CoA-carboxylase gene from colza); ADH (alcohol dehydrogenase I gene from maize); bar (phosphinothricin-N-acetyltransferases gene from *Streptomyces hygroscopicus*); CP4-EPSPS (5-enolpyruvylshikimate-3-phosphate synthase gene from *Agrobacterium tumefaciens* strain); Cry (gene encoding the *Bacillus thuringiensis δ*-endotoxin); IVR (invertase gene from maize); LEC (lectin gene from soybean); nptII (neomycin phosphotransferase II gene); p35S (promoter of the 35 S cauliflower mosaic virus); pat (phosphinothricin-N-acetyltransferases gene from *Streptomyces viridochromogenes*); pFMV (promoter of the figwort mosaic virus); phy (phytase gene from maize); PLD (phospholipase D gene from rice); pNOS (promoter of the nopaline synthase gene); tNOS (terminator of the nopaline synthase gene); uidA (*β*-glucuronidase).

**Table 9:** Representative examples illustrating DNA walking strategies targeting GMO.

DNA walking approaches	Characterized regions	Targets	References
Restriction-based methods			

Inverse PCR	Transgene flanking regions	Bt11	[92, 93]

Cassette PCR	Transgene flanking regions	GTS40-3-2	[94]
	Transgene flanking regions	GT73	[95]
	Transgene flanking regions	MON1445	[96]
	Transgene flanking regions	TC-19	[97]
	Transgene flanking regions	TT51-1	[98]
	Transgene flanking regions	KeFeng-6	[99]
	Transgene flanking regions	KeFeng-8	[100]
	Transgene flanking regions	B73-6-1	[101]
	Transgene flanking regions	B72-8-11	[102]
	Transgene flanking regions	B72-8-11b	[103]
	Transgene flanking regions	LY038	[104]
	Transgene flanking regions	MON89788	[104]
	Transgene flanking regions	3272	[104]
	Transgene flanking regions and unnatural element associations	CHB-351	[105, 106]
	Transgene flanking regions and unnatural element associations	Bt176	[95, 106]
	Transgene flanking regions and unnatural element associations	GA21	[95, 106]
	Transgene flanking regions and unnatural element associations	Bt11	[95, 106]
	Transgene flanking regions and unnatural element associations	T25	[106, 107]
	Transgene flanking regions and unnatural element associations	MON810	[106, 108]
	Transgene flanking regions and unnatural element associations	DAS-59122-7	[104, 106]
	Unnatural element associations	MON88017	[106]
	Unnatural element associations	MON863 × NK603	[106]
	Unnatural element associations Unnatural element associations	MON863 × NK603 × MON810	[106]
	Unnatural element associations	NK603	[106]
	Unnatural element associations	MON863	[106]

Extension-based methods			

LT-RADE	Transgene flanking regions and unnatural element associations	MON810	[109, 110]
	Transgene flanking regions and unnatural element associations	LLRICE62	[109, 110]
	Transgene flanking regions and unnatural element associations	T45	[110]
	Transgene flanking regions and unnatural element associations	A2704-12	[110]
	Transgene flanking regions and unnatural element associations	LLCOTTON25	[110]

PCR-based methods			

TAIL-PCR	Transgene flanking regions	MON863	[111, 112]
	Transgene flanking regions	KeFeng-6	[113]
	Transgene flanking regions	B73-6-1	[114]

SiteFinding PCR	Transgene flanking regions	KMD1	[115]
	Unnatural element associations	MIR162	[116]

APAgene GOLD Genome Walking Kit	Transgene flanking regions and unnatural element associations	Bt rice	[33, 117, 118]
	Transgene flanking regions and unnatural element associations	MON863	[118]

**Table 10:** Representative examples illustrating NGS strategies targeting GMO.

NGS strategies	NGS platforms	Targets	Target sizes	References
Targeted sequencing	HiSeq (Illumina)	vip3Aa2 from MIR162	150 bp to 2 Kbp	[116]
	PacBio RS (Pacific Biosciences)	vip3Aa2 from MIR162	150 bp to 2 Kbp	[116]
	454 system (Roche Applied Science)	ssIIb	157 bp	[119]
	454 system (Roche Applied Science)	Bt11 gene	324 bp	[119]
	454 system (Roche Applied Science)	Bt176 gene	206 bp	[119]
	454 system (Roche Applied Science)	LEC	118 bp	[119]
	454 system (Roche Applied Science)	p35S/CTP4	171 bp	[119]
	454 system (Roche Applied Science)	CP4-EPSPS	498 bp	[119]
	454 system (Roche Applied Science)	p35S	195 bp	[119]
	454 system (Roche Applied Science)	tNOS	180 bp	[119]

Whole genome sequencing	HiSeq (Illumina)	MON17903 soybean	1115 Mbp	[120]
	HiSeq (Illumina)	MON87704 soybean	1115 Mbp	[120]
	HiSeq (Illumina)	FP967 flax	373 Mbp	[121]
	HiSeq (Illumina)	LLRICE62 rice	385 Mbp	[122]
	HiSeq (Illumina)	TT51-1 rice	385 Mbp	[123]
	HiSeq (Illumina)	T1c-19 rice	385 Mbp	[123]
	HiSeq (Illumina)	Bt rice	385 Mbp	[124]

CP4-EPSPS (5-enolpyruvylshikimate-3-phosphate synthase gene from *Agrobacterium tumefaciens* strain); CTP4 (chloroplast transit peptide 4 from the *Arabidopsis thaliana* epsps gene); LEC (lectin gene from soybean); p35S (promoter of the 35 S cauliflower mosaic virus); ssIIb (starch synthase IIb gene from maize); tNOS (terminator of the nopaline synthase gene); VIP3A (vegetative insecticidal protein 3A).
